# A Multicenter Clinical Study To Demonstrate the Diagnostic Accuracy of the GenMark Dx ePlex Blood Culture Identification Gram-Negative Panel

**DOI:** 10.1128/JCM.02484-20

**Published:** 2021-08-18

**Authors:** Donna M. Wolk, Stephen Young, Natalie N. Whitfield, Jennifer L. Reid, Adam Thornberg, Karen C. Carroll, Blake W. Buchan, Thomas E. Davis, Hossein Salimnia

**Affiliations:** a Geisinger Health, Danville, Pennsylvania, USA; b TriCore Reference Laboratories, Albuquerque, New Mexico, USA; c GenMark Diagnostics, Carlsbad, California, USA; d Johns Hopkins University School of Medicine, Baltimore, Maryland, USA; e Medical College of Wisconsin, Milwaukee, Wisconsin, USA; f Indiana University, Bloomington, Indiana, USA; g Detroit Medical Center, Detroit, Michigan, USA; Washington University School of Medicine

**Keywords:** ePlex, GenMark, Gram-negative, bacteremia, rapid diagnostics, AST, antimicrobial testing

## Abstract

Bacteremia can progress to septic shock and death without appropriate medical intervention. Increasing evidence supports the role of molecular diagnostic panels in reducing the clinical impact of these infections through rapid identification of the infecting organism and associated antimicrobial resistance genes. We report the results of a multicenter clinical study assessing the performance of the GenMark Dx ePlex investigational-use-only blood culture identification Gram-negative panel (BCID-GN), a rapid diagnostic assay for detection of bloodstream pathogens in positive blood culture (PBC) bottles. Prospective, retrospective, and contrived samples were tested. Results from the BCID-GN were compared to standard-of-care bacterial identification methods. Antimicrobial resistance genes (ARGs) were identified using PCR and sequence analysis. The final BCID-GN analysis included 2,444 PBC samples, of which 926 were clinical samples with negative Gram stain results. Of these, 109 samples had false-negative and/or -positive results, resulting in an overall sample accuracy of 88.2% (817/926). After discordant resolution, overall sample accuracy increased to 92.9% (860/926). Pre- and postdiscordant resolution sample accuracy excludes 37 Gram-negative organisms representing 20 uncommon genera, 10 Gram-positive organisms, and 1 *Candida* species present in 5% of samples that are not targeted by the BCID-GN. The overall weighted positive percent agreement (PPA), which averages the individual PPAs from the 27 targets (Gram-negative and ARG), was 94.9%. The limit of detection ranged from 10^4^ to 10^7^ CFU/ml, except for one strain of Fusobacterium necrophorum at 10^8^ CFU/ml.

## INTRODUCTION

Extrapolating from data obtained in high-income countries, Fleischmann et al. (1) estimated that the annual global burden of sepsis is about 35.1 million cases, with approximately 5.3 million deaths. The mortality rate in patients with severe sepsis and septic shock increases by 7.4% for each hour that appropriate antibiotic therapy is delayed (2). Circumstances like these necessitate immediate initiation of empirical broad-spectrum antibiotics for all patients with presumed infection (3) until the organism is definitively identified and its susceptibility is determined (4). The administration of broad-spectrum antibiotics can lead to complications, including toxicity, increased antibiotic resistance, and Clostridioides difficile toxin-related diseases. Thus, it is essential to determine the nature of the infecting organism(s) and corresponding antibiotic susceptibilities as soon as possible to allow the selection of the appropriate and targeted therapy.

Currently, there are several commercially available, FDA-cleared blood culture identification (BCID) systems that use molecular methods for rapid identification of pathogens and antibiotic resistance genes (ARGs) in positive blood culture (PBC) bottles. One such system offers a single panel to detect 43 Gram-positive (GP) and Gram-negative (GN) bacteria and yeasts as well as 10 antibiotic resistance markers (5–7). Two other platforms offer separate panels for the identification of GP and GN bacteria, but neither has a fungal panel (8, 9). While other commercial assays provide multitarget pathogen panels, few of the existing assays incorporate “Pan” targets into their panel design. Lack of a Pan target adds to reliance on accurate interpretation of the primary Gram stain result for selection of the correct test panel or could result in failure to recognize the presence of an off-panel organism or an unsuspected mixed infection. The BCID-GN is a comprehensive panel comprising 21 specific Gram-negative genus or species targets, including anaerobic bacteria and less common aerobic targets. In addition, BCID-GN detects 6 genetic markers of antibiotic resistance as well as Pan targets for select Gram-positive bacteria and *Candida* species to aid in detection of polymicrobial cultures.

The purpose of this study was to establish the analytical and clinical performance characteristics of the GenMark Dx ePlex investigational-use-only (IUO) blood culture identification Gram-negative panel (BCID-GN) to support the regulatory premarket submission. We report the results of a study in which six large clinical microbiology laboratories assessed the performance of BCID-GN on 2,444 prospective, retrospective, and contrived samples.

## MATERIALS AND METHODS

### Study population.

The study population included patients of all ages and genders, with 10% of samples from patients <18 years of age (see Table S1 in the supplemental material). PBCs were collected for standard patient care and diagnosis in 10 geographically diverse regions within the United States, including the following cities: Albuquerque, NM; Baltimore, MD; Charleston, SC; Danville, PA; Detroit, MI (two sites); Harvey, IL; Indianapolis, IN; Milwaukee, WI; and San Diego, CA. Samples were obtained from clinical and reference laboratories representing a variety of clinical settings, including outpatient clinics, hospitals, emergency departments, extended care facilities, and any other facilities where patients sought medical care.

### Overall study design and conduct.

Clinical samples were cultured and tested as ordered per the standard-of-care procedures (SOC) at each site. The study was performed using two protocols, one for collection and a second for collection and testing; both were approved by a central Institutional Review Board (IRB) (Quorum, Seattle, WA) and/or the site-specific IRB.

### Sample collection and storage.

Thirteen different blood culture bottle types (i.e., broth) from three manufacturers (Becton, Dickinson, bioMérieux, Inc., and Thermo Fisher Scientific) were used. Clinical sample inclusion criteria were defined as residual inoculated blood culture bottles, which were flagged as positive in an automated continuous monitoring blood culture system with a readable Gram stain result exhibiting Gram-negative organisms. Samples were tested when prospective (fresh or frozen), within the time frame specified by the manufacturer (10), or retrospective (frozen blood culture broth samples from trial sites). Contrived samples were prepared in BD Bactec blood culture bottles (Plus Aerobic/F, Plus Anaerobic/F, Lytic/10 Anaerobic/F, or Peds Plus/F) as previously described (11–14). For the prospective collection, exclusion criteria were defined as any fresh broth from positive blood culture bottle(s) from a patient that was already tested. (In the retrospective arm, two bottles from the same patient may have been tested if different types flagged positive [i.e., one anaerobic and one aerobic]). Bottles containing charcoal, and samples with insufficient volume to complete required testing were also excluded.

### The GenMark Dx ePlex BCID-GN panel testing.

Samples were tested at one of six clinical sites with the BCID-GN (10). Frozen samples were labeled such that testing personnel were blinded to the sample origin (i.e., prospective, retrospective, and contrived) and the expected organism results (10). Each day of testing, negative and positive controls were tested using the BCID-GN. Controls were prepared by GenMark Dx, and testing rotated among different instrument bays with each day of testing to ensure all bays were targeted for equal testing during the study.

The BCID-GN device is a sample-to-answer cartridge that uses electrowetting and eSensor technology for the extraction, amplification, and competitive DNA hybridization (15, 16) for the identification of 21 bacterial targets, six ARGs associated with extended-spectrum beta-lactamases (ESBL) and carbapenemases, and two Pan targets direct from PBC bottles (Table 1). The BCID-GN ARG assays detect the following genetic determinants of resistance: CTX-M, KPC, IMP, VIM, NDM, and OXA (OXA-23 and OXA-48). Results for ARG targets are only reported when an associated organism known to carry the gene is detected by the BCID-GN (Table 2).

**TABLE 1:** ePlex blood culture identification Gram-negative panel

Target type	Target
Bacterial targets	Acinetobacter baumannii
	Bacteroides fragilis
	*Citrobacter* spp.
	Cronobacter sakazakii
	Enterobacter cloacae complex
	Enterobacter (non*-cloacae* complex)
	Escherichia coli
	Fusobacterium necrophorum
	Fusobacterium nucleatum
	Haemophilus influenzae
	Klebsiella oxytoca
	Klebsiella pneumoniae group^*a*^
	Morganella morganii
	Neisseria meningitidis
	Proteus spp.
	Proteus mirabilis
	Pseudomonas aeruginosa
	Salmonella spp.
	*Serratia* spp.
	Serratia marcescens
	Stenotrophomonas maltophilia
Antimicrobial resistance markers	CTX-M (*bla*_CTX-M_)
	IMP (*bla*_IMP_)
	KPC (*bla*_KPC_)
	NDM (*bla*_NDM_)
	OXA (*bla*_OXA_)^*b*^
	VIM (*bla*_VIM_)
Pan targets	Pan-Gram-positive^*c*^
	Pan-*Candida*^*d*^

a K. pneumoniae, *K. quasipneumoniae*, and *K. variicola*.

b 23 and 48 like.

c Coverage of the Pan Gram-positive target includes the following: Bacillus cereus group (including B. cereus and B. thuringiensis), Bacillus subtilis group (including *B. amyloliquefaciens*, B. atrophaeus, B. licheniformis, and B. subtilis*)*, *Enterococcus* (including *E. avium*, *E. casseliflavus*, *E. cecorum*, *E. dispar*, *E. durans*, E. faecalis, E. faecium, *E. gallinarum*, *E. hirae*, *E. italicus*, *E. malodoratus*, *E. pseudoavium*, *E. raffinosus*, *E. saccharolyticus*, and *E. sanguinicola*), Staphylococcus (including *S. arlettae*, S. aureus, *S. auricularis*, *S. capitis*, *S. caprae*, *S. carnosus*, *S. chromogenes*, *S. cohnii*, S. epidermidis, *S. gallinarum*, *S. haemolyticus*, S. hominis, *S. hyicus*, S. intermedius, S. lentus, *S. lugdunensis*, *S. muscae*, *S. pasteurii*, *S. pettenkoferi*, S. pseudintermedius, *S. saccharolyticus*, *S. saprophyticus*, *S. schleiferi*, S. sciuri, *S. simulans*, *S. vitulinus*, *S. warneri*, *and S. xylosus*), and/or Streptococcus (including S. agalactiae, *S. anginosus*, S. bovis, *S. constellatus*, *S. cricetid*, S. dysgalactiae, S. equi, S. equinus, S. gallolyticus, S. gordonii, *S. infantarius*, *S. infantis*, S. intermedius, S. mitis, S. oralis, *S. parasanguinis*, *S. peroris*, S. pneumoniae, S. pyogenes, *S. salivarius*, S. sanguinis, and *S. thoraltensis)*.

d Coverage of the Pan *Candida* target includes C. albicans, C. glabrata, C. krusei, and C. parapsilosis.

**TABLE 2:** Resistance marker and organism combinations reported by BCID-GN panel

Organism(s)	Resistance marker^*a*^					
	CTX-M	IMP	KPC	NDM	OXA	VIM
Acinetobacter baumannii, Citrobacter spp., Enterobacter cloacae complex, Enterobacter (non-cloacae complex), Escherichia coli, Klebsiella oxytoca, Klebsiella pneumoniae group, Morganella morganii, Proteus spp., Proteus mirabilis, Pseudomonas aeruginosa, Salmonella, Serratia spp., Serratia marcescens	X	X	X	X	X	X
Bacteroides fragilis, Fusobacterium necrophorum, Fusobacterium nucleatum, Haemophilus influenzae, Neisseria meningitidis						
Cronobacter sakazakii			X			
Stenotrophomonas maltophilia	X					

a An “X” denotes that the genetic target is detected for that organism list.

Briefly, after inverting the blood culture bottle several times to mix, 50 μl was aspirated and loaded into the loading port of the BCID-GN cartridge, and the cap was depressed to close the port. Each cartridge was barcoded according to the manufacturer’s instructions, the barcode was scanned by the ePlex instrument, and the cartridge was inserted into an available bay. Upon completion, the ePlex instrument ejected the cartridge for disposal and a BCID-GN report was generated (10).

### Reference and comparator methods.

The comparator method(s) for organism identification were the site’s SOC, including traditional culture, FDA-cleared matrix-assisted laser desorption ionization–time-of-flight mass spectrometry (MALDI-TOF MS) (i.e., bioMérieux Vitek MS and Bruker Biotyper), and automated phenotypic identification and antibiotic susceptibility platforms (e.g., Becton, Dickinson [BD] Phoenix, bioMérieux Vitek 2, and Siemens MicroScan). The phenotypic methods listed above varied at different sites and served as the site’s reference identification standard. Due to issues with organism misidentification, samples with a member of the Acinetobacter calcoaceticus*-baumannii* complex (15) or Candida parapsilosis (16) identified by SOC were confirmed using analytically validated PCR amplification assays followed by bidirectional sequencing (PCR/sequencing) or 16S sequencing by Laboratory Corporation of America Holdings (LabCorp [Burlington, NC]) according to FDA clinical trial instructions outlined in the 510k summary (10). The comparator methods for ARGs were analytically validated by real-time PCR amplification assay(s) followed by bidirectional sequencing, developed and performed by LabCorp.

### Discordant resolution method.

Results from collected samples that were discordant between the BCID-GN and the comparator method(s) (i.e., false negative, false positive) were tested with analytically validated PCR amplification assay(s) followed by bidirectional sequencing by LabCorp to determine the presence or absence of the organism.

### Data and statistical methods.

For each target on the BCID-GN, investigational results were compared to the comparator method results. Organisms or ARGs detected by both investigational and comparator methods were classified as true positive, whereas those detected by neither were classified as true negative. Organisms or ARGs detected by comparator methods but not the BCID-GN were classified as false negative, whereas organisms or ARGs detected by the BCID-GN but not by comparator methods were classified as false positive.

Positive percent agreement (PPA) and negative percent agreement (NPA) with comparator method results were determined for each target on the BCID-GN, according to standard laboratory calculations (17). The two-sided 95% confidence intervals (CI) were calculated for PPA and NPA. For ARGs, performance was calculated including only samples with associated organisms detected by comparator methods.

## RESULTS

### Total samples.

A combination of 354 prospectively enrolled, 1,326 retrospectively selected (from frozen, banked PBC samples), and 780 contrived (isolates spiked into whole blood and blood culture bottles) samples were tested with the BCID-GN. Of the 354 prospective samples, 171 were collected and tested (fresh never frozen), while 183 were collected and frozen for later testing. One of these samples was withdrawn due to organism identification by unacceptable methods. Four additional samples lacking final, valid BCID-GN results were withdrawn and excluded from analysis (one prospective, three retrospective). Of the 2,460 total samples initially tested with the BCID-GN, 126 yielded invalid results for an initial validity rate of 94.9%. After repeat testing, 12 samples were excluded as nonevaluable, since they continued to produce invalid results; the final validity rate was 99.5%. Therefore, the final analysis included 926 clinical samples with Gram-negative Gram stain results (349 prospective, 577 retrospective) and 777 contrived samples evaluable for the organism-specific targets present on the BCID-GN. An additional 741 clinical samples without Gram-negative Gram stain results were evaluated for the Pan targets only.

### Demographic/sample information.

Subject demographic information was linked to the evaluable prospectively and retrospectively collected samples with Gram-negative stain results (*n* = 349 and 577, respectively) and is provided in Table S1 in the supplemental material. Mean and median ages of the two groups were 59.2 and 62.0, respectively, for prospectively collected samples and 59.6 and 64.0 for retrospectively collected samples. Female gender was higher in the prospective samples (51.9%) but lower in the retrospective samples (46.8%).

Approximately 84% of clinical samples were tested fresh or frozen within 12 h of bottle positivity in ultralow temperature freezers for several months before testing with the investigational assay. Testing of the remaining clinical samples occurred at the following intervals: 7% within 12 to 24 h; 4% within 24 to 48 h; 4% within 48 to 96 h; <1% within 96 to 168 h; and <1% within 168 to 504 h (10 retrospective samples positive for a *Candida* species). All testing of contrived samples occurred within 2 h of the bottle flag; samples identified after the 2-h period were excluded. No difference in performance was observed due to storage conditions (10). The instructions for use (IFU) allow for room temperature storage up to 7 days, refrigerated storage up to 1 month, and storage at −80°C to −20°C for up to 18 months after bottle positivity (10).

Three commercially available blood culture systems were included in the study during a time when no relevant background DNA or recalls were reported from the manufacturers. Across all sites, BD Bactec blood culture bottles represented the majority (84% across all categories, 6% of which were pediatric bottles), with the remaining 16% split among BacT/Alert (9%) and TREK (7%). Of the total pediatric bottles, 39% were clinical samples and the remaining were contrived samples. The distribution of bottle types is shown in Table S2.

### Distribution of standard laboratory procedures.

In some cases, comparator methods differed between the fresh and frozen samples tested due to changes in the site’s standard methods utilized at the time of original testing. The majority (79.6%) of prospective fresh samples grew organisms identified by FDA-cleared MALDI-TOF mass spectrometry (Vitek MS or Bruker). Prospective frozen samples contained organisms identified primarily by Vitek 2 (63.7%) or MicroScan (25.3%). Retrospective Gram-negative samples contained organisms identified by Phoenix (52.5%) or Vitek 2 (31.5%). Although some nonviable amplification of microbial DNA has been previously reported (18), during the time of the ePlex study, no background amplification signals were identified.

### Assay performance.

There were 926 prospective and retrospective PBC samples with Gram stains that displayed Gram-negative organisms, including 842 samples with only Gram-negative bacilli. There were 67 samples containing mixtures of Gram-negative and Gram-positive bacteria, two containing mixtures of Gram-negative bacteria and yeast, four with mixed Gram-negative and Gram-positive bacteria and yeast, and 11 with Gram-variable organisms.

**FIG 1:**
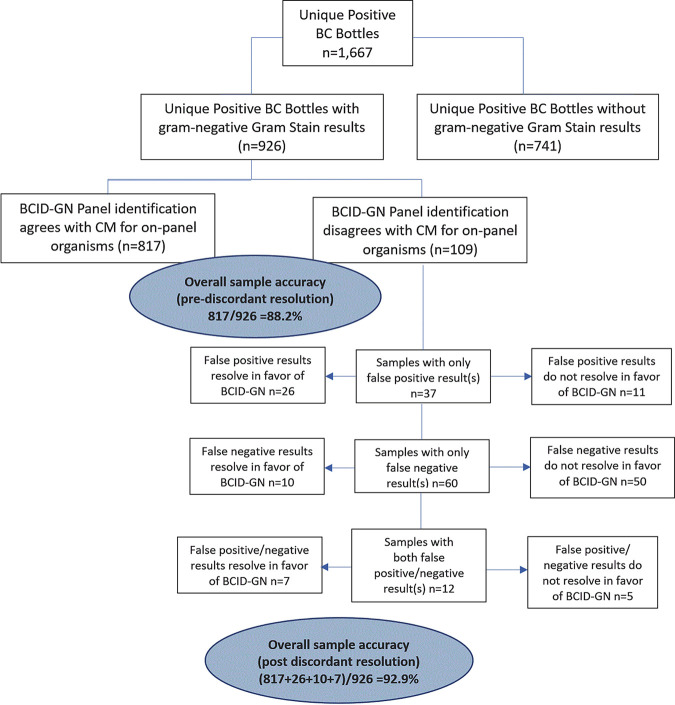
Sample analysis flow diagram describes the categorization of sample results used for calculations of accuracy.

A total of 72 samples had one or more false-negative results, but the BCID-GN correctly identified the organisms and ARGs identified by comparator methods in 92.2% (854/926) of these samples. There were 109 samples with false-negative and/or -positive results, resulting in an overall sample accuracy where the BCID-GN detected all organisms/ARGs found by comparator methods and did not detect additional organisms/ARGs (i.e., false positive) of 88.2% (817/926). After discordant resolution of the false-negative and -positive results (i.e., correction for organisms/ARGs confirmed by other test methods to agree with the BCID-GN results), the overall sample accuracy increased to 92.9% (860/926). The above-described sample results are depicted in Fig. 1. These results exclude 37 Gram-negative organisms representing 20 uncommon genera, 10 Gram-positive organisms, and one *Candida* species, detected in 5% of samples that are not targeted by the BCID-GN.

The PPA and NPA for the BCID-GN targets versus comparator methods are provided in Table 3 for the 13 Gram-negative targets in the order *Enterobacterales*, Table 4 for the eight non-*Enterobacterales* Gram-negative targets, Table 5 for the two Pan targets, and Table 6 for the six ARG targets. The overall weighted PPA includes all sample types and represents an average of the individual PPAs; the overall PPA was 93.6% across all 29 targets and 94.9% excluding the two Pan targets. For all tables, results are presented in the following categories: clinical samples (both prospectively and retrospectively collected samples), contrived samples, and all samples combined. Footnotes are provided, by target, to indicate the number of false-negative or false-positive results that were resolved by discordant resolution using PCR/sequencing. The results presented were calculated before discordant resolution and represent a comparison to comparator methods (e.g., local laboratory results). Results in parentheses indicate 95% confidence intervals.

**TABLE 3:** BCID-GN panel *Enterobacterales* target clinical performance

Target	Clinical samples				Contrived samples				Combined samples			
	PPA		NPA		PPA		NPA		PPA		NPA	
	TP/TP+FN	% (95% CI)	TN/TN+FP	% (95% CI)	TP/TP+FN	% (95% CI)	TN/TN+FP	% (95% CI)	TP/TP+FN	% (95% CI)	TN/TN+FP	% (95% CI)
*Citrobacter* spp.^*a*^	25/26	96.2 (81.1–99.3)	896/900	99.6 (98.9–99.8)	43/43	100 (91.8–100)	734/734	100 (99.5–100)	68/69	98.6 (92.2–99.7)	1,630/1,634	99.8 (99.4–99.9)
C. sakazakii	1/1	100 (20.7–100)	925/925	100 (99.6–100)	45/45	100 (92.1–100)	732/732	100 (99.5–100)	46/46	100 (92.3–100)	1,657/1,657	100 (99.8–100)
Enterobacter spp.^*b*^	20/22	90.9 (72.2–97.5)	903/904	99.9 (99.4–100)	36/36	100 (90.4–100)	741/741	100 (99.5–100)	56/58	96.6 (88.3–99.0)	1,644/1,645	99.9 (99.7–100)
E. cloacae complex^*c*^	66/69	95.7 (88.0–98.5)	852/857	99.4 (98.6–99.8)	35/37	94.6 (82.3–98.5)	739/740	99.9 (99.2–100)	101/106	95.3 (89.4–98.0)	1,591/1,597	99.6 (99.2–99.8)
E. coli ^*d*^	263/273	96.3 (93.4–98.0)	650/653	99.5 (98.7–99.8)	52/52	100 (93.1–100)	725/725	100 (99.5–100)	315/325	96.9 (94.4–98.3)	1,375/1,378	99.8 (99.4–99.9)
K. oxytoca ^*e*^	40/47	85.1 (72.3–92.6)	876/879	99.7 (99.0–99.9)	20/20	100 (83.9–100)	757/757	100 (99.5–100)	60/67	89.6 (80.0–94.8)	1,633/1,636	99.8 (99.5–99.9)
K. pneumoniae group^*f*^	164/169	97.0 (93.3–98.7)	753/757	99.5 (98.6–99.8)	72/72	100 (94.9–100)	705/705	100 (99.5–100)	236/241	97.9 (95.2–99.1)	1,458/1,462	99.7 (99.3–99.9)
M. morganii ^*g*^	13/13	100 (77.2–100)	912/913	99.9 (99.4–100)	49/49	100 (92.7–100)	728/728	100 (99.5–100)	62/62	100 (94.2–100)	1,640/1,641	99.9 (99.7–100)
Proteus spp.	76/78	97.4 (91.1–99.3)	848/848	100 (99.5–100)	9/9	100 (70.1–100)	768/768	100 (99.5–100)	85/87	97.7 (92.0–99.4)	1,616/1,616	100 (99.8–100)
P. mirabilis	72/74	97.3 (90.7–99.3)	852/852	100 (99.6–100)	9/9	100 (70.1–100)	768/768	100 (99.5–100)	81/83	97.6 (91.6–99.3)	1,620/1,620	100 (99.8–100)
Salmonella ^*h*^	20/21	95.2 (77.3–99.2)	905/905	100 (99.6–100)	34/35	97.1 (85.5–99.5)	742/742	100 (99.5–100)	54/56	96.4 (87.9–99.0)	1,647/1,647	100 (99.8–100)
*Serratia*	44/44	100 (92.0–100)	881/882	99.9 (99.4–100)	36/36	100 (90.4–100)	741/741	100 (99.5–100)	80/80	100 (95.4–100)	1,622/1,623	99.9 (99.7–100)
S. marcescens ^*i*^	43/43	100 (91.8–100)	882/883	99.9 (99.4–100)	19/19	100 (83.2–100)	758/758	100 (99.5–100)	62/62	100 (94.2–100)	1,640/1,641	99.9 (99.7–100)

a Citrobacter braakii (2) and Citrobacter freundii (2) were detected in 4/4 false-positive clinical samples using PCR/sequencing.

b A species of the Enterobacter non-*cloacae* complex was not detected in 2 false-negative clinical samples, but PCR/sequencing detected E. cloacae, which was not identified by standard laboratory procedures. Enterobacter non-*cloacae* complex was not detected in the false-positive sample using PCR/sequencing.

c A species of the Enterobacter cloacae complex was not detected in 1 false-negative clinical sample, but PCR/sequencing and MALDI-TOF detected E. coli, which was not identified by standard laboratory procedures. E. cloacae was detected in 2/5 false-positive clinical samples using PCR/sequencing.

d E. coli was not detected in 1 false-negative clinical sample using PCR/sequencing; a different organism was not detected. E. coli was detected in 3/3 false-positive clinical samples using PCR/sequencing.

e K. oxytoca was not detected in 2 false-negative clinical samples using PCR/sequencing, but 16S sequencing detected Raoultella ornithinolytica and *R. planticola*, which were not identified by standard laboratory procedures. K. oxytoca was not detected in an additional 2 false-negative clinical samples using PCR/sequencing; a different organism was not detected. Klebsiella oxytoca was detected in 3/3 false-positive clinical samples using PCR/sequencing.

f K. pneumoniae was not detected in 1 false-negative clinical sample, but PCR/sequencing and MALDI-TOF detected K. oxytoca, which was not identified by standard laboratory procedures. K. pneumoniae was not detected in an additional 2 false-negative clinical samples using PCR/sequencing; a different organism was not detected. K. pneumoniae was detected in 4/4 false-positive clinical samples using PCR/sequencing.

g M. morganii was detected in 1/1 false-positive clinical sample using PCR/sequencing.

h Salmonella was not detected in 1 false-negative clinical sample, but PCR/sequencing detected E. coli, which was not identified by standard laboratory procedures.

i S. marcescens was detected in the one false-positive clinical sample using PCR/sequencing.

**TABLE 4:** BCID-GN panel non-*Enterobacterales* targets clinical performance

Target	Clinical samples				Contrived samples				Combined samples			
	PPA		NPA		PPA		NPA		PPA		NPA	
	TP/TP+FN	% (95% CI)	TN/TN+FP	% (95% CI)	TP/TP+FN	% (95% CI)	TN/TN+FP	% (95% CI)	TP/TP+FN	% (95% CI)	TN/TN+FP	% (95% CI)
A. baumannii ^*a*^	19/19	100 (83.2–100)	905/906	99.9 (99.4–100)	55/55	100 (93.5–100)	722/722	100 (99.5–100)	74/74	100 (95.1–100)	1,627/1,628	99.9 (99.7–100)
B. fragilis ^*b*^	25/28	89.3 (72.8–96.3)	896/898	99.8 (99.2–99.9)	40/40	100 (91.2–100)	737/737	100 (99.5–100)	65/68	95.6 (87.8–98.5)	1,633/1,635	99.9 (99.6–100)
F. necrophorum	1/1	100 (20.7–100)	925/925	100 (99.6–100)	47/48	97.9 (89.1–99.6)	729/729	100 (99.5–100)	48/49	98.0 (89.3–99.6)	1,654/1,654	100 (99.8–100)
F. nucleatum ^*c*^	5/5	100 (56.6–100)	920/921	99.9 (99.4–100)	47/47	100 (92.4–100)	730/730	100 (99.5–100)	52/52	100 (93.1–100)	1,650/1,651	99.9 (99.7–100)
H. influenzae	14/14	100 (78.5–100)	912/912	100 (99.6–100)	41/41	100 (91.4–100)	736/736	100 (99.5–100)	55/55	100 (93.5–100)	1,648/1,648	100 (99.8–100)
N. meningitidis ^*d*^	0/0		925/926	99.9 (99.4–100)	44/44	100 (92.0–100)	733/733	100 (99.5–100)	44/44	100 (92.0–100)	1,658/1,659	99.9 (99.7–100)
P. aeruginosa ^*e*^	83/88	94.3 (87.4–97.5)	834/838	99.5 (98.8–99.8)	32/32	100 (89.3–100)	745/745	100 (99.5–100)	115/120	95.8 (90.6–98.2)	1,579/1,583	99.7 (99.4–99.9)
S. maltophilia ^*f*^	11/14	78.6 (52.4–92.4)	911/912	99.9 (99.4–100)	36/36	100 (90.4–100)	741/741	100 (99.5–100)	47/50	94.0 (83.8–97.9)	1,652/1,653	99.9 (99.7–100)

a A. baumannii was detected in the 1/1 false-positive clinical sample using PCR/sequencing.

b B. fragilis was not detected in 2 false-negative clinical samples, but PCR/sequencing detected *B. caccae* and B. thetaiotaomicron, which were not identified by standard laboratory procedures. B. fragilis was not detected in the additional 1 false-negative clinical sample using PCR/sequencing, but PCR/sequencing detected F. nucleatum (F. nucleatum was also detected by the ePlex BCID-GN panel). B. fragilis was detected in 2/2 false-positive clinical samples using PCR/sequencing.

c F. nucleatum was detected in 1/1 false-positive clinical sample using PCR/sequencing.

d N. meningitidis was not detected in the one false-positive clinical sample using PCR/sequencing.

e P. aeruginosa was detected in 2/4 false-positive clinical samples using PCR/sequencing.

f S. maltophilia was detected in the one false-positive clinical sample using PCR/sequencing.

**TABLE 5:** BCID-GN panel Pan target clinical performance

Target	Clinical samples				Contrived samples				Combined samples			
	PPA		NPA		PPA		NPA		PPA		NPA	
	TP/TP+FN	% (95% CI)	TN/TN+FP	% (95% CI)	TP/TP+FN	% (95% CI)	TN/TN+FP	% (95% CI)	TP/TP+FN	% (95% CI)	TN/TN+FP	% (95% CI)
Pan *Candida* spp.^*a*^	104/110	94.5 (88.6–97.5)	1554/1557	99.8 (99.4–99.9)	0/0		777/777	100 (99.5–100)	104/110	94.5 (88.6–97.5)	2,331/2,334	99.9 (99.6–100)
Pan Gram-positive^*b*^	628/649	96.8 (95.1–97.9)	996/1018	97.8 (96.7–98.6)	0/0		776/777	99.9 (99.3–100)	628/649	96.8 (95.1–97.9)	1,772/1,795	98.7 (98.1–99.1)

a Candida albicans (2 samples) and Candida glabrata (1 sample) were detected in 3/3 false-positive clinical samples using PCR/sequencing.

b *Bacillus* (the Gram-positive organism identified by standard laboratory procedures) was not detected in 2 false-negative clinical samples using PCR/sequencing, but 16S sequencing detected Paenibacillus lautus and Paenibacillus urinalis, which were not identified by standard laboratory procedures. The Gram-positive organism identified by standard laboratory procedures (i.e., *Bacillus* and S. aureus) was not detected using PCR/sequencing in an additional 2 false-negative clinical samples; a different organism was not detected. *Bacillus* (1 sample), *Enterococcus* (3 samples), Staphylococcus (4 samples), and Streptococcus (8 samples) were detected in 16/22 false-positive clinical samples using PCR/sequencing.

**TABLE 6:** BCID-GN panel antimicrobial resistance markers clinical performance

Target	Clinical samples				Contrived samples				Combined samples			
	PPA		NPA		PPA		NPA		PPA		NPA	
	TP/TP+FN	% (95% CI)	TN/TN+FP	% (95% CI)	TP/TP+FN	% (95% CI)	TN/TN+FP	% (95% CI)	TP/TP+FN	% (95% CI)	TN/TN+FP	% (95% CI)
CTX-M^*a*^	74/85	87.1 (78.3–92.6)	754/754	100 (99.5–100)	75/75	100 (95.1–100)	437/437	100 (99.1–100)	149/160	93.1 (88.1–96.1)	1,191/1,191	100 (99.7–100)
IMP	0/0		829/829	100 (99.5–100)	40/40	100 (91.2–100)	436/436	100 (99.1–100)	40/40	100 (91.2–100)	1,265/1,265	100 (99.7–100)
KPC	7/8	87.5 (52.9–97.8)	821/822	99.9 (99.3–100)	44/44	100 (92.0–100)	477/477	100 (99.2–100)	51/52	98.1 (89.9–99.7)	1,298/1,299	99.9 (99.6–100)
NDM	0/0		829/829	100 (99.5–100)	54/54	100 (93.4–100)	422/422	100 (99.1–100)	54/54	100 (93.4–100)	1,251/1,251	100 (99.7–100)
OXA^*b*^	10/13	76.9 (49.7–91.8)	814/816	99.8 (99.1–99.9)	37/37	100 (90.6–100)	439/439	100 (99.1–100)	47/50	94.0 (83.8–97.9)	1,253/1,255	99.8 (99.4–100)
VIM	0/0		829/829	100 (99.5–100)	42/42	100 (91.6–100)	434/434	100 (99.1–100)	42/42	100 (91.6–100)	1,263/1,263	100 (99.7–100)

a For 8/11 remaining false-negative CTX-M target detections, discordant analysis was not evaluable due to sample contamination.

b In 1/3 false-negative clinical samples, the OXA signal was above the threshold for detection; however, an associated organism was not detected by the BCID-GN, and the OXA target was reported as NA. One additional false-negative sample was tested with an FDA-cleared multiplex assay, and OXA was not detected. The isolate from the remaining false-negative sample tested negative for OXA-23 and OXA-48 by qPCR.

The BCID-GN was tested to establish limits of detection (LOD) at bacterial suspensions with densities between 1 × 10^4^ and 1 × 10^7^ CFU/ml. The density range was selected to represent the typical densities of bacteria encountered at the time of bottles being flagged positive by blood culture instruments and to create standard microbial densities for analysis. One exception was F. necrophorum, which was tested at a density of 1 × 10^7^ and 1 × 10^8^ CFU/ml due to its slower metabolism; it was known that 1 × 10^8^ CFU/ml was required.

### (i) *Enterobacterales*.

The overall PPA for the common *Enterobacterales* ranged from a high of 100% for Cronobacter sakazakii, Morganella morganii, Serratia marcescens, and *Serratia* spp. to a low of 89.6% for Klebsiella oxytoca. Overall NPA for the common *Enterobacterales* ranged from 99.6% to 100%. The combined sample size for each target in Table 3 is 1,703. For positive results, the sample size ranged from a low of 46 for C. sakazakii to a high of 325 for Escherichia coli. Across the *Enterobacterales* targets, there were 22 false-positive results from retrospective (frozen) and prospective (fresh) clinical samples. Based on the discordant resolution with PCR/sequencing, 18 of these were confirmed to be correctly detected by the BCID-GN. *Enterobacterales* targets with discordant results are discussed below.

*(a) Citrobacter species*. Overall PPA and NPA were 98.6% and 99.8%, respectively, with one false-negative and four false-positive results. Citrobacter braakii (*n* = 2) and Citrobacter freundii (*n* = 2) were detected using PCR/sequencing in the four false-positive samples.

*(b) Enterobacter species*. Overall PPA and NPA for Enterobacter (non-*cloacae* complex) were 96.6% and 99.9%, respectively, with two false-negative and one false-positive result. In two false-negative samples, PCR/sequencing detected E. cloacae instead of a non-*cloacae* species. For E. cloacae complex, overall PPA and NPA were 95.3% and 99.6%, respectively, with five false negative and six false positives, of which two false-positive samples had E. cloacae detected by PCR/sequencing (the same two samples that had false-negative results for the Enterobacter target).

*(c) Escherichia coli*. Overall PPA and NPA of E. coli were 96.9% and 99.8%, with 10 false-negative and three false-positive results. The presence of E. coli was confirmed in all three false-positive samples by PCR or sequencing.

*(d) Klebsiella oxytoca and K. pneumoniae group*. The K. oxytoca target’s overall PPA was 89.6% and NPA was 99.8%, with seven false-negative and three false-positive results. In two false-negative samples, a *Raoultella* species was detected by 16S sequencing instead of K. oxytoca, and in two other false-negative samples, K. oxytoca was not detected by PCR/sequencing. K. oxytoca was detected by PCR/sequencing in all three false-positive samples. The K. pneumoniae group target had an overall PPA of 97.9% and NPA of 99.7%, with five false-negative and four false-positive results. One of the false-negative results was determined to be K. oxytoca by both PCR/sequencing and MALDI-TOF MS (one of the samples that had false-positive K. oxytoca results, discussed above), with K. pneumoniae not detected in two of the remaining false-negative samples. All four false-positive samples had the presence of K. pneumoniae confirmed by PCR, MALDI-TOF, or sequencing.

*(e) Morganella morganii*. The M. morganii target had an overall PPA of 100% and NPA of 99.9%, with one false-positive result that was confirmed to be M. morganii by PCR/sequencing.

*(f) Proteus species*. The Proteus species and Proteus mirabilis targets had similar results, with an overall PPA of 97.6% to 97.7% and NPA of 100%. There were two false-negative results, both the result of one sample; both had P. mirabilis detected by PCR/sequencing.

*(g) Salmonella species*. The Salmonella species target had an overall PPA of 96.4% and NPA of 100%, with two false-negative results, one of which was determined to be E. coli by PCR/sequencing.

*(h) Serratia species*. The *Serratia* spp. and S. marcescens targets both had an overall PPA of 100% and NPA of 99.9%, with one false-positive result that was confirmed to be S. marcescens by PCR/sequencing.

### (ii) Non-*Enterobacterales*.

The overall PPA for the non-*Enterobacterales* ranged from a high of 100% for A. baumannii, Fusobacterium nucleatum, Haemophilus influenzae, and Neisseria meningitidis to a low of 94.0% for Stenotrophomonas maltophilia. Overall NPA for the non-*Enterobacterales* ranged from 99.7% to 100%. The combined sample size for each target is in Table 4. Non-*Enterobacterales* is 1,703 for all targets except for A. baumannii complex, for which one sample could not be verified as A. baumannii or another species in the complex (*n* = 1,702). For positive results, the sample size ranged from a low of 44 for N. meningitidis to a high of 120 for Pseudomonas aeruginosa. Across the non-*Enterobacterales* targets, there were 10 false-positive results in clinical samples; seven of these were confirmed to be correctly detected by the BCID-GN based on discordant resolution with PCR/sequencing. Non-*Enterobacterales* targets with discordant results are discussed below.

*(a) Acinetobacter baumannii.* The A. baumannii target had an overall PPA of 100% and NPA of 99.9%, with one false-positive result that was confirmed as A. baumannii by PCR/sequencing.

*(b) Bacteroides fragilis.* The B. fragilis target had an overall PPA of 95.6% and NPA of 99.9%, with three false-negative and two false-positive results. In two of the false-negative samples, PCR/sequencing detected Bacteroides caccae and Bacteroides thetaiotaomicron. Both false-positive samples had B. fragilis detected by PCR/sequencing.

*(c) Fusobacterium.*Fusobacterium necrophorum had an overall PPA of 98.0% and NPA of 100%, with one false-negative result. F. nucleatum had an overall PPA of 100% and NPA of 99.9%, with one false-positive sample that had F. nucleatum detected by PCR/sequencing.

*(d) Neisseria meningitidis.* The N. meningitidis target had 100% overall PPA and 99.9% NPA, with one false-positive result that was not detected by PCR/sequencing.

*(e) Pseudomonas aeruginosa.* The P. aeruginosa target had an overall PPA of 95.8% and NPA of 99.7%, with five false-negative results and four false-positive results. The five false-negative samples and two of the four false-positive samples had P. aeruginosa detected by PCR/sequencing.

*(f) Stenotrophomonas maltophilia.* The S. maltophilia target had an overall PPA of 94.0% and NPA of 99.9%, with three false-negative results and one false-positive result. Two false-negative and one false-positive sample had S. maltophilia detected by PCR/sequencing.

### (iii) Pan targets.

The combined performance data for Pan targets is represented in Table 5 for 2,444 samples. The Pan *Candida* target’s PPA and NPA were 94.5% and 99.9%, respectively (*n* = 110 for positive samples), while the Pan Gram-positive target had 96.8% PPA and 98.7% NPA (*n* = 649 for positive samples). In terms of false-negative discrepancies, *Bacillus* spp. identified by standard laboratory procedures were not detected in two clinical samples; instead, 16S sequencing detected Paenibacillus lautus and Paenibacillus urinalis. There were three false-positive Pan *Candida* results, all of which were confirmed to be correctly detected by the BCID-GN after discordant resolution (Candida albicans [*n* = 2], Candida glabrata [*n* = 1]). There were 22 false-positive Pan Gram-positive results in clinical samples; 16 were confirmed to be correctly detected by the BCID-GN based on discordant resolution with PCR/sequencing (*Bacillus* spp. [*n* = 1], *Enterococcus* spp. [*n* = 3], Staphylococcus spp. [*n* = 4], and Streptococcus spp. [*n* = 8]).

### (iv) ARGs.

Table 6 represents performance for ARG targets associated with the microbes listed in Table 2. An associated organism for CTX-M or KPC was present in 1,351 samples, and for the remaining ARGs, an associated organism was present in 1,305 samples. IMP, NDM, and VIM targets had 100% PPA and NPA but were only detected in 40, 54, and 42 contrived samples, respectively. Including clinical and contrived samples, KPC PPA was 98.1% and NPA was 99.9% (8 clinical and 44 contrived positive samples). The CTX-M target represents the lowest combined PPA at 93.1%, with 100% NPA. For 8/11 remaining false-negative CTX-M target detections, discordant analysis was not evaluable due to sample contamination. With those samples excluded, the CTX-M PPA is 97.4%. Additionally, three false-negative CTX-M results occurred due to the associated genetic target not being detected by the BCID-GN (CTX-M signal was above the threshold for detection but was reported as not applicable [NA] due to the lack of organism detection). OXA PPA was 94.0% and NPA was 99.8% (13 clinical and 37 contrived samples). An investigation into false-negative results similar to that undertaken for CTX-M determined that one OXA-positive sample was contaminated. One sample also did not have the associated organism detected by the BCID-GN, but the OXA signal was above the threshold for detection. For ARG comparison with phenotype results, refer to the 510k summary (10).

### (v) Microbial diversity.

Table S3 summarizes data from Tables 1 to 4, substratified by organism and organism group. This view visualizes the breadth and depth of the microbial diversity tested in the clinical study. Seventy-nine species or microbial groups had targets detected in the samples tested. Forty-three Gram-positive microbes were detected by the Pan Gram-positive target and four yeast species were detected by the Pan *Candida* target.

Table S4 summarizes results from the rare microbes identified by standard of care methods that are off-panel for the BCID-GN. For prospective and retrospective samples, these include 10 Gram-positive organisms representing seven genera, 37 Gram-negative organisms representing 19 genera, and one yeast, Candida lusitaniae. The most common genera not included on the BCID-GN were Acinetobacter (*n* = 6), *Providencia* (*n* = 6), and *Achromobacter* (*n* = 3), with species from the genera *Aeromonas*, *Bacteroides*, *Clostridium*, *Moraxella*, *Micrococcus*, *Leclercia*, and *Sphingomonas* each identified in two samples.

### (vi) Polymicrobial samples.

Table 7 summarizes clinical samples with multiple organisms detected by the BCID-GN. The BCID-GN identified 103 samples with multiple organisms. Of these, 84 (9.0%) samples contained two organisms, and 19 (2.0%) samples had three organisms. Within the 103 total codetections, 69 contained the same organisms identified by comparator methods; the remaining 34 codetections (polymicrobial samples) included 39 organisms that were missed by the comparator method (i.e., false positive). Discordant resolution confirmed the presence of 32 organisms, whereas four were unconfirmed and three with Pan Gram-positive false-positive results were not tested.

**TABLE 7:** Summary of polymicrobial codetections by the BCID-GN and discrepancies with comparator methods (clinical samples)^*b*^

Codetection type	No. of codetections (% of samples)	No. of discrepant codetections	No. of discrepant organism ARG(s)^*a*^
Total	103 (11.1%)	34	39
Double detections	84 (9.0%)	22	22
Triple detections	19 (2.0%)	12	17

a A discrepant organism/ARG is one that was detected by the BCID-GN panel but not by the comparator method(s) (i.e., false positive); 36/39 false-positive organisms were investigated using PCR/sequencing (3 Pan GP organisms were not tested). Of the 36 false-positive organisms tested, 32 were detected, 3 were not detected, and 1 was indeterminate.

b Refer to the 510k summary (Tables 60 and 61) for more detail (10).

Table 8 represents the 103 distinct multiple organisms and antibiotic gene combinations detected by the BCID-GN. The 32 discrepant false-positive organisms that were confirmed present in the sample by discordant resolution are denoted with an asterisk (*), and the remaining seven discrepant false-positive organisms are denoted with a caret (^). The 38 additional distinct codetections identified by comparator methods, which include false-negative organism(s), are provided in Table 9.

**TABLE 8:** Distinct multiple organism/antibiotic resistance gene combinations detected by the BCID-GN and discrepancies with comparator methods (clinical samples)

Distinct codetection combinations detected by the BCID-GN panel^*b*^				
1	2	3	ARG	No. of samples (no. discrepant^*a*^)
A. baumannii*	K. pneumoniae GP*	Pan GP*	CTX-M, OXA	1 (1)
A. baumannii	Pan GP			4 (0)
A. baumannii	Pan GP^		OXA	4 (1)
B. fragilis*	E. cloacae complex	Pan GP		1 (1)
B. fragilis*	E. coli			2 (1)
B. fragilis	Pan GP			1 (0)
*Citrobacter*	E. cloacae complex^			1 (1)
Citrobacter*	E. cloacae complex*	K. oxytoca		2 (2)
*Citrobacter*	E. coli			1 (0)
Citrobacter*	K. oxytoca			1 (1)
Citrobacter*	K. oxytoca	K. pneumoniae GP		1 (1)
*Citrobacter*	K. oxytoca*	K. pneumoniae GP		1 (1)
*Citrobacter*	K. pneumoniae GP			1 (0)
*Citrobacter*	K. pneumoniae GP	Pan GP	CTX-M	1 (0)
*Citrobacter*	M. morganii*	Pan GP		1 (1)
*Citrobacter*	P. mirabilis	Pan GP*		1 (1)
*Citrobacter*	Pan GP*			3 (2)
E. cloacae complex	E. coli	K. pneumoniae GP		1 (0)
E. cloacae complex	K. pneumoniae GP			1 (0)
E. cloacae complex	P. aeruginosa*	Pan GP		1 (1)
E. cloacae complex	Pan Candida*			1 (1)
E. cloacae complex	Pan *Candida*	Pan GP		1 (0)
E. cloacae complex	Pan GP^			4 (1)
E. coli	K. oxytoca*			3 (1)
E. coli	K. oxytoca	Pan GP		1 (0)
E. coli	K. pneumoniae GP			2 (0)
E. coli*	K. pneumoniae GP		CTX-M	1 (1)
E. coli	M. morganii			1 (0)
E. coli	P. mirabilis			3 (0)
E. coli	P. mirabilis	Pan GP		1 (0)
E. coli	Pan GP*			10 (3)
E. coli	Pan GP		CTX-M	1 (0)
Enterobacter *^*	K. pneumoniae GP			1 (1)
Enterobacter	Pan *Candida*			1 (0)
Enterobacter	Pan GP			1 (0)
H. influenzae	N. meningitidis^	P. aeruginosa^		1 (1)
K. oxytoca	K. pneumoniae GP*			2 (1)
K. oxytoca	Pan GP*			4 (2)
K. oxytoca	S. marcescens*			2 (1)
K. pneumoniae GP	P. mirabilis			1 (0)
K. pneumoniae GP	Pan GP*			6 (1)
K. pneumoniae GP	Pan GP^		CTX-M, KPC	1 (1)
K. pneumoniae GP*	Pan GP	S. marcescens		1 (1)
K. pneumoniae GP	S. maltophilia			1 (0)
M. morganii	P. aeruginosa*	Pan GP		1 (1)
M. morganii	P. mirabilis			2 (0)
M. morganii	Pan GP	Proteus		1 (0)
P. aeruginosa	P. mirabilis	Pan GP		1 (0)
P. aeruginosa	Pan GP			2 (0)
P. mirabilis	Pan GP*			8 (2)
P. mirabilis	Pan GP		CTX-M	1 (0)
Pan *Candida*	Pan GP			2 (0)
Pan GP	S. maltophilia			1 (0)
Pan GP	S. marcescens			3 (0)

a A discrepant organism/ARG is one that was detected by the BCID-GN but not by the comparator method(s) (i.e., false positive); 36/39 false-positive organisms were investigated using PCR/sequencing (3 Pan GP organisms were not tested). Of the 36 false-positive organisms tested, 32 were detected, 3 were not detected, and 1 was indeterminate.

b *, Discordant false-positive organism confirmed present by PCR/sequencing; ^, discordant false-positive organism not confirmed present by PCR/sequencing.

**TABLE 9:** Additional codetections identified by the comparator methods (clinical samples) for coinfections with false-negative organisms only

Distinct organism/antibiotic resistance gene combinations detected by comparator methods^*b*^					No. of samples (no. discreptant)	No. of discrepant organism(s)/ARG(s)^*a*^
1	2	3	4	ARG		
Aeromonas caviae*	E. coli	Enterococcus casseliflavus	K. oxytoca		1 (1)	*E. casseliflavus* (1)
Aeromonas veronii*	E. cloacae				1 (1)	E. cloacae (1)
C. albicans	E. faecium	Staphylococcus hominis			1 (1)	C. albicans (1)
C. albicans	P. aeruginosa				1 (1)	C. albicans (1)
*C. braakii*	E. cloacae	K. oxytoca			1 (1)	*C. braakii* (1), K. oxytoca (1)
C. freundii	*Enterococcus*				1 (1)	*Enterococcus* (1)
C. glabrata	E. aerogenes	Staphylococcus			1 (1)	Staphylococcus (1)
C. glabrata	P. mirabilis				1 (1)	C. glabrata (1)
C. krusei	S. epidermidis				1 (1)	S. epidermidis (1)
*C. youngae*	K. oxytoca				1 (1)	K. oxytoca (1)
E. aerogenes	K. oxytoca	Leclercia adecarboxylata*			2 (2)	E. aerogenes (2)
E. aerogenes	P. aeruginosa				1 (1)	P. aeruginosa (1)
E. cloacae	E. coli				1 (1)	E. coli (1)
E. cloacae	E. faecalis				1 (1)	E. faecalis (1)
E. cloacae	M. morganii				1 (1)	E. cloacae (1)
E. cloacae	S. maltophilia				1 (1)	S. maltophilia (1)
E. coli	E. faecalis	K. pneumoniae			1 (1)	E. coli (1)
E. coli	E. faecium			CTX-M	1 (1)	E. faecium (1)
E. coli	K. pneumoniae				1 (1)	E. coli (1)
E. coli	P. aeruginosa				1 (1)	P. aeruginosa (1)
E. coli	P. mirabilis				1 (1)	E. coli (1)
E. coli	P. mirabilis	P. vulgaris	Streptococcus viridans group		1 (1)	S. viridans group (1)
E. coli	P. mirabilis	Providencia stuartii*	*S. anginosus* GP	CTX-M	1 (1)	E. coli (1)
E. coli	*S. anginosus* GP				1 (1)	*S. anginosus* gp (1)
E. faecalis	K. pneumoniae				2 (2)	E. faecalis (1), K. pneumoniae (1)
E. faecalis	M. morganii	P. mirabilis			1 (1)	E. faecalis (1)
E. faecalis	P. aeruginosa	S. aureus			1 (1)	P. aeruginosa (1)
E. faecalis	S. maltophilia				1 (1)	E. faecalis (1)
K. pneumoniae	P. aeruginosa				1 (1)	P. aeruginosa (1)
K. pneumoniae	S. aureus				1 (1)	S. aureus (1)
K. pneumoniae	Staphylococcus				1 (1)	Staphylococcus (1)
K. pneumoniae	Staphylococcus haemolyticus	Nonfermenting GN bacilli*			1 (1)	K. pneumoniae (1)
P. aeruginosa	S. maltophilia				2 (2)	S. maltophilia (2)
P. mirabilis	Providencia stuartii*				1 (1)	P. mirabilis (1)
S. maltophilia	Streptococcus				1 (1)	Streptococcus (1)

a A discrepant organism/ARG is defined as one that was detected by the comparator method(s), which should have been detected by the BCID-GN but was not (excludes organisms not targeted by the BCID-GN).

b *, Off-panel organism not targeted by the BCID-GN.

Table 9 illustrates the distribution of false-negative results found in mixtures for the BCID-GN. Seven codetections include a BCID-GN off-panel organism, denoted with an asterisk, which is not expected to be identified by the BCID-GN. Other false-negative results include a variety of microbes in mixtures, some rapid growing and some slow growing. No apparent pattern of microbial false-negative results is evident. E. coli, P. aeruginosa, and Enterococcus faecalis were the most frequently missed pathogens, followed by C. albicans.

## DISCUSSION

The BCID-GN provides results describing pathogens identified in PBC bottles in approximately 90 min on the ePlex platform, a scalable (3 to 24 bays), random, and continuous-access instrument with automated quality control monitoring. With this system, hands-on time from PBC bottles is <2 min (19). The panel was challenged with a well-populated sample set representing the most identified Gram-negative bloodstream pathogens (20–25) and ARG targets, with an overall accuracy of 92.9% after discordant resolution. Although molecular discordant resolution has limitations, it is a practical option for a comparative accuracy study for molecular multiplex methods, as conventional culture methods cannot detect nucleic acids and, therefore, cannot verify the presence or absence of genetic sequences. This approach has been used in similar previously published studies (11–14).

The panel is diverse and reflects common bloodstream pathogens as well as rare microbes with high pathogenicity. For example, as Salmonella strains become more resistant (26), a rapid Salmonella species result can be useful in streamlining antimicrobial treatment. Another example is the non-*Enterobacterales*; although less common than the *Enterobacterales* targets, they are pathogens that pose significant public health risks (24, 25, 27). Both H. influenzae and N. meningitidis are commonly reportable to state public health laboratories (28). For N. meningitidis, public health and infection prevention investigations can include contacts with the source patient; therefore, rapid results support these efforts and could speed targeted antimicrobial therapy. Antimicrobial chemoprophylaxis of close contacts of a patient with meningococcal disease is important to prevent secondary cases (29), regardless of whether a meningococcal outbreak is suspected.

BCID-GN utility is also noted for the identification of bacteremia caused by two *Fusobacterium* spp., which are associated with a high mortality rate for patients with renal insufficiency, heart failure or malignancies, and Lemierre’s syndrome (30). Rapid identification of *Fusobacterium* species and B. fragilis supports the ability to broaden coverage for anaerobic bacteria, which can be absent in some empirical antibiotic regimens (30, 31). Finally, identification of the nonfermenters P. aeruginosa, S. maltophilia, and A. baumannii will support the escalation of therapy while broadening coverage to include the multidrug resistance (MDR) of these bacteria (32–35). The BCID-GN can provide information to hospital epidemiologists, infection preventionists, and bed management teams by providing faster identification of MDR microbes, upon which to base isolation practices for microbes with the potential for health care-associated outbreaks (36–38).

As with any molecular syndromic test panel, it is impossible to include all microbes that could be identified by routine culture methods in a panel. In this case, 5% of organisms were identified by culture only. The breadth of the culture-positive/BCID-GN-negative samples includes 39 rare species, five of which could be opportunistic pathogens from skin contamination of blood cultures in the proper clinical setting. Only Providencia stuartii (*n* = 6) exceeded five samples, followed by Acinetobacter *radioresistans* (*n* = 3) and then Micrococcus luteus and Leclercia adecarboxylata (*n* = 2 each). The remaining 35 species were represented by a frequency of one among 926 samples tested (0.01%); nine Gram-positive and 33 Gram-negative microbes needed to be identified by traditional methods. In comparison, some alternative commercial panels have fewer targets, thereby missing clinically significant organisms, such as anaerobes (*Bacteroides* spp. and *Fusobacterium* spp.) (8) or S. maltophilia, whereby awareness of its predictable and intrinsic antibiotic resistance adds benefit to therapeutic decision-making. The added diversity of pathogens detected by BCID-GN adds value compared to some other methods (8), which do not provide the extensive diversity, sensitivity, or improved ability to discern mixtures (39, 40); however, newer versions of panels are now available with a broader microbe menu (6). One option for identification of a much broader diversity of microbes is the use of MALDI-TOF MS methods, direct from blood cultures (41–43). MALDI-TOF MS does not yet provide any resistance profiles for Gram-positive or Gram-negative organisms and does not work well for mixed infections.

The BCID-GN is the only currently available, FDA-cleared molecular panel that incorporates a Pan Gram-positive and Pan *Candida* targets into a Gram-negative panel. The inclusion of these targets could limit the conditions under which Gram-positive or fungal antimicrobial coverage is inadvertently discontinued if the Gram stain is not sensitive enough to visualize these microbes or when fast-growing Gram-negative microbes predominate at the time of the stain. These targets are an important addition to antimicrobial decision strategy, because coinfections are not uncommon and the added protection of the Pan Gram-positive call prevents improper deescalation (40, 44). The use of the Pan Gram-positive target limits the number of times one would have to test both panels, as one would do in some other methods (45), with potential cost-benefits. The Accelerate Pheno reports a panbacterial target, but the result is not Gram stain specific; it simply alerts the user to the presence of bacteria (46).

The Pan Gram-positive target is designed to primarily detect organisms that may be missed by Gram stain or are potentially Gram variable. The Pan Gram-positive results include organisms commonly considered contaminants found on the skin that may also be opportunistic pathogens and associated with illnesses such as endocarditis. While the lack of inclusion of other skin contaminants (*C. acnes*, *Lactobacillus* spp., *Micrococcus* spp.) in the Pan Gram-positive result might be useful in certain populations (47), in other populations, laboratorians might need to scrutinize patient history more closely (48). Some of the organisms detected by the pan-GP target could be BCC (13, 49, 50) (refer to Table S3), and some could be true infections. Traditional chart reviews to assess blood culture contamination events still apply to molecular methods.

Performance of the full ePlex BCID Gram-positive panel for immunocompromised patients, transplant patients, cancer patients, and others likely exhibiting a significant infection (48) may be necessary. For example, implications for *Bacillus* spp. that are associated with severe infections in transplant populations (51), Staphylococcus epidermidis, *Micrococcus* spp., and Cutibacterium acnes (51, 52), which may be implicated in endocarditis and other infections, and *Corynebacterium* spp., which can cause MDR infections due to Corynebacterium jeikeium and Corynebacterium striatum (51). The Pan *Candida* target on the BCID-GN only detects C. albicans, C. parapsilosis, C. glabrata, and C. krusei, while other, less prevalent *Candida* species, including C. auris, are not included. When yeast species are observed on Gram stain or when a Pan *Candida* target is identified in the absence of a Gram stain exhibiting yeast, the BCID-FP (fungal pathogen) panel could be used for further testing (53).

One of the most important advantages of the BCID-GN is its ability to differentiate among microbes with similar Gram reactions, thereby increasing its ability to identify coinfections. The BCID-GN identified 103 samples with multiple (two or three) organisms (11.1%). Importantly, 34 codetections included 39 organisms that were missed by the comparator method, with 32 organisms confirmed present by discordant resolution. Of 103 multiple distinct organisms and antibiotic gene combinations detected by the BCID-GN, a Pan Gram-positive result was detected in 69 coinfections; for 15 of these (22%), the SOC method did not detect the Gram-positive organism. Of the 15, 12 were confirmed, and three were not tested. The Gram stain result only identified the presence of Gram-positive microbes in one of 15 samples, missing 14/15 Gram-positive results. A positive result for BCID-GN where the culture did not grow an organism could be the result of a patient being treated with antibiotics that could render an organism nonculturable but present and viable in the bloodstream, or it could represent the short-lived presence of nucleic acid sequence in the bloodstream, which could be detected by molecular methods.

The BCID-GN method is a PCR that was tested to establish limits of detection (LOD) at bacterial suspensions with densities between 1 × 10^4^ and 1 × 10^7^ CFU/ml, with the exception of F. necrophorum, which was tested at a density of 1 × 10^8^ CFU/ml. The density range was selected to represent the typical densities of bacteria encountered at the time of bottles being flagged positive by blood culture instruments.

The LODs for microbes on the BCID-GN support detection of mixed cultures, as evidenced by the larger number of mixed infections identified in this trial compared to others (54). The low LOD becomes important with mixed infections, since some slower-growing microbes may be present in lower numbers than the faster-growing species. Because background DNA (i.e., the presence of DNA from nonviable organisms) has been found in various brands of blood culture media and has been detected by several molecular methods (55, 56), it is considered best practice to correlate results from rapid molecular detection with subsequent subculture growth and the prescribed antimicrobials present in the patient’s bloodstream at the time of blood culture collection. With any rapid molecular detection method, including BCID-GN, vigilance and investigation of rare discrepancies caused by dead microbes or excess DNA in blood culture broth should occur (18).

In this study, some coinfections were identified solely by SOC methods; they represent a mixture of microbes similar to those that were missed by SOC. A plausible assumption is that sampling variability and sampling error contributed, as defined by the statistical Poisson distribution characterizing microbes present in solution at low density, a known limitation to any method that attempts to identify microbes in low density (57). There are no apparent trends to the microbes undetected by the BCID-GN; we refer the reader to the 510k summary for further information (10). Therefore, the results likely indicate the presence of organisms in lower concentrations at the time of testing. E. coli, P. aeruginosa, and E. faecalis were the most commonly missed pathogens, followed by C. albicans, which is relatively slow growing.

The BCID-GN contains targets for the class A carbapenemase, KPC, the class B metallo-beta-lactamases IMP, VIM, and NDM, and the class D beta-lactamase OXA (23 and 48), frequently produced by A. baumannii; all confer resistance to carbapenems (58). The BCID-GN targets the CTX-M group of ESBLs, which are the most common ESBLs globally and mediate resistance to penicillins, cephalosporins, and monobactams (59). Accuracy for antimicrobial resistance gene targets (*n* = 1,351) was high, ranging from 93.1% to 100%, and had matching performance that is similar to or better than those of phenotypic and molecular comparator methods for antimicrobial susceptibility testing (60). The opportunity for antimicrobial stewardship programs to escalate therapy has been clearly demonstrated for the identification of resistance genes in Gram-negative bacteria causing bloodstream infections (61). Likewise, in local pockets where MDR clones are common, NPAs for ceftriaxone susceptibility in E. coli and K. pneumoniae in the absence of either CTX-M or a carbapenemase gene were 98% and 93 to 94%, respectively (40), suggesting that, depending on local epidemiology, formulary, and stewardship practices, the absence of a resistance gene can guide antimicrobial deescalation. Negative results for these select antimicrobial resistance gene assays do not indicate susceptibility, as there are multiple mechanisms of resistance (62).

One limitation of the BCID-GN is the exclusion of the *mcr-1* and *mcr-2* genes, which are indicative of colistin resistance (63). In the United States, colistin resistance is relatively rare and colistin is not a first-line antibiotic; therefore, reflex testing can occur. However, colistin resistance is a global challenge, and its panel exclusion may represent a challenge in some global settings (64).

The overall study limitations include potential bias introduced by testing more than one bottle from the same patient in the retrospective arm and the need to use contrived samples, as they may not reflect the contemporary bacterial strains circulating in the local geography.

While it is common to use contrived samples in clinical trials to supplement the breadth of the microbes and ARG targets that occur during the clinical trial period, it is prudent to verify multiplex methods with as many fresh samples as possible and to comply with state and federal regulations for new method verification. Overall, the targeted study population and sample matrices mimic reality in many laboratories; thus, results should be generalizable to other settings. The proportion of prospective or retrospective clinical results was acceptable to the U.S. FDA. The testing was performed in geographically distinct regions of the United States, with populations ranging from community-based hospitals to quaternary care health care organizations, performed by medical technologists or equivalents. As with any molecular blood culture identification method, clinical presentation and the use of additional laboratory testing of PBCs is still required (e.g., subculturing to identify organisms that are not detected by the panels for susceptibility testing, differentiation of mixed growth, and association of antimicrobial resistance marker genes to a specific organism) for the final diagnosis of bacterial bloodstream infection.

The ePlex BCID-GN offers advantages as a significant aid in the diagnosis of specific agents of bacteremia. The Pan target calls are present to alert laboratory scientists to a potential error in Gram stain reading and microbes that may be present at low density, and all laboratories can benefit from the ability to detect anaerobic pathogens and a wider range of ARG genes. The throughput range of the ePlex instrument is practical for both community hospitals and large core laboratories, with particular advantages to integrated delivery laboratories that can match throughput needs to the size of the individual laboratories while maintaining the same protocols and computer interfaces. With the addition of the BCID-GN to the previously FDA-cleared ePlex BCID panels, including the blood culture identification Gram-positive panel (BCID-GP) (13) and fungal pathogen panel (BCID-FP) (14), the ePlex BCID panels become the most comprehensive rapid molecular diagnostic offering for the identification of pathogens and resistance mechanisms causing bloodstream infections, with the ability to detect a total of 41 bacterial targets, 15 fungal targets, and 10 ARG markers from a PBC bottle.
