# Supplementary material for: A Multicenter Clinical Study To Demonstrate the Diagnostic Accuracy of the GenMark Dx ePlex Blood Culture Identification Gram-Negative Panel
Source: J Clin Microbiol. 2021 Aug 18;59(9):e02484-20. doi: 10.1128/JCM.02484-20 (PMC8373019; doi:10.1128/JCM.02484-20)
Supplement: Supplemental file 1 — Tables S1 to S4. Download JCM.02484-20-s0001.pdf, PDF file, 0.3 MB [file jcm.02484-20-s0001.pdf]

**Supplemental Table 1. Subject Demographics by Collection Type**

| | Prospectively Collected (N=349) | Retrospectively Collected (N=577) |
|---|---|---|
| <1 yr. | 7 (2.0) | 9 (1.6) |
| 1-17 yrs. | 10 (2.9) | 20 (3.5) |
| 18-44 yrs. | 50 (14.3) | 78 (13.5) |
| 45-64 yrs. | 124 (35.5) | 193 (33.4) |
| 65-84 yrs. | 125 (35.8) | 226 (39.2) |
| 85+ yrs. | 33 (9.5) | 49 (8.5) |
| Unknown | --- | 2 (0.3) |

## Supplemental Table 2. Blood Culture Bottle Type*

| | All (N=2444) n (%) | Prospective Fresh (N=167) n (%) | Prospective Frozen (N=182) n (%) | Retrospective (N=577) n (%) | Retrospective Pan Only (N=741) n (%) | Contrived (N=777) n (%) |
|---|---|---|---|---|---|---|
| BacT/Alert - SA (Standard Aerobic) | 130 (5.3) | 0 (0.0) | 37 (20.3) | 5 (0.9) | 88 (11.9) | 0 (0.0) |
| BacT/Alert - SN (Standard Anaerobic) | 90 (3.7) | 0 (0.0) | 20 (11.0) | 8 (1.4) | 62 (8.4) | 0 (0.0) |
| BACTEC - Lytic/10 Anaerobic/F | 517 (21.2) | 51 (30.5) | 44 (24.2) | 144 (25.0) | 150 (20.2) | 128 (16.5) |
| BACTEC - PLUS Aerobic/F | 1147 (46.9) | 65 (38.9) | 54 (29.7) | 197 (34.1) | 285 (38.5) | 546 (70.3) |
| BACTEC - PLUS Anaerobic/F | 68 (2.8) | 10 (6.0) | 6 (3.3) | 17 (2.9) | 21 (2.8) | 14 (1.8) |
| BACTEC - Peds Plus /F | 145 (5.9) | 3 (1.8) | 6 (3.3) | 21 (3.6) | 26 (3.5) | 89 (11.5) |
| BACTEC - Standard/10 Aerobic/F | 117 (4.8) | 4 (2.4) | 6 (3.3) | 60 (10.4) | 47 (6.3) | 0 (0.0) |
| BACTEC - Standard/10 Anaerobic/F | 23 (0.9) | 5 (3.0) | 2 (1.1) | 7 (1.2) | 9 (1.2) | 0 (0.0) |
| BACTEC - Standard Anaerobic/F | 36 (1.5) | 1 (0.6) | 7 (3.8) | 20 (3.5) | 8 (1.1) | 0 (0.0) |
| VersaTREK - Redox 1 | 102 (4.2) | 25 (15.0) | 0 (0.0) | 50 (8.7) | 27 (3.6) | 0 (0.0) |
| VersaTREK - Redox 1 EZ Draw | 39 (1.6) | 0 (0.0) | 0 (0.0) | 25 (4.3) | 14 (1.9) | 0 (0.0) |
| VersaTREK - Redox 2 | 24 (1.0) | 3 (1.8) | 0 (0.0) | 17 (2.9) | 4 (0.5) | 0 (0.0) |
| VersaTREK - Redox 2 EZ Draw | 6 (0.2) | 0 (0.0) | 0 (0.0) | 6 (1.0) | 0 (0.0) | 0 (0.0) |

| | All | Prospective Fresh | Prospective Frozen | Retrospective | Retrospective Pan Only | Contrived |
|---|---|---|---|---|---|---|
| | (N=2444) | (N=167) | (N=182) | (N=577) | (N=741) | (N=777) |
| | n (%) | n (%) | n (%) | n (%) | n (%) | n (%) |

***Based on analysis of stored sample testing during the study, no meaningful difference was observed for room temperature storage up to seven days, refrigerated storage up to one month, and at -80˚C to -20˚C for up to 18 months after bottle positivity, as listed in the published FDA product summary.**

## Supplement Table 3: PPA for Species Detected in Genus and Group Assays

| Target Species or Groups Detected by Comparator Method | PPA | | | | | |
|---|---|---|---|---|---|---|
| | Clinical Samples | | Contrived Samples | | Combined Samples | |
| | TP/TP+F N | % (95% CI) | TP/TP+F N | % (95% CI) | TP/TP+F N | % (95% CI) |
| ***Citrobacter* spp.** | **20/21** | **95.2 (77.3-99.2)** | **43/43** | **100 (91.8-100)** | **68/69** | **98.6 (92.2-99.7)** |
| *Citrobacter braakii* | 2/3 | 66.7 (20.8-93.9) | 8/8 | 100 (67.6-100) | 10/11 | 90.9 (62.3-98.4) |
| *Citrobacter freundii* | 13/13 | 100 (77.2-100) | 27/27 | 100 (87.5-100) | 44/44 | 100 (92.0-100) |
| *Citrobacter koseri* | 4/4 | 100 (51.0-100) | 4/4 | 100 (51.0-100) | 9/9 | 100 (70.1-100) |
| *Citrobacter youngae* | 1/1 | 100 (20.7-100) | 4/4 | 100 (51.0-100) | 5/5 | 100 (56.6-100) |
| ***Enterobacter* (non-*cloacae* complex)** | **12/12** | **100 (75.8-100)** | **36/36** | **100 (90.4-100)** | **56/58** | **96.6 (88.3-99.0)** |
| *Enterobacter aerogenes* | 12/12 | 100 (75.8-100) | 26/26 | 100 (87.1-100) | 45/47 | 95.7 (85.8-98.8) |
| *Enterobacter amnigenus* | | | 10/10 | 100 (72.2-100) | 10/10 | 100 (72.2-100) |
| *Enterobacter gergoviae* | | | | | 1/1 | 100 (20.7-100) |
| ***Enterobacter cloacae* complex** | **47/50** | **94.0 (83.8-97.9)** | **35/37** | **94.6 (82.3-98.5)** | **101/106** | **95.3 (89.4-98.0)** |
| *Enterobacter asburiae* | | | 6/8 | 75.0 (40.9-92.9) | 6/8 | 75.0 (40.9-92.9) |
| *Enterobacter cloacae* | 46/49 | 93.9 (83.5-97.9) | 28/28 | 100 (87.9-100) | 93/96 | 96.9 (91.2-98.9) |
| *Enterobacter hormaechei* | 1/1 | 100 (20.7-100) | 1/1 | 100 (20.7-100) | 2/2 | 100 (34.2-100) |

| Target Species or Groups Detected by Comparator Method | PPA | | | | | |
|---|---|---|---|---|---|---|
| | Clinical Samples | | Contrived Samples | | Combined Samples | |
| | TP/TP+F N | % (95% CI) | TP/TP+F N | % (95% CI) | TP/TP+F N | % (95% CI) |
| ***Proteus* spp.** | **54/55** | **98.2 (90.4-99.7)** | **9/9** | **100 (70.1-100)** | **85/87** | **97.7 (92.0-99.4)** |
| *Proteus mirabilis* | 50/51 | 98.0 (89.7-99.7) | 9/9 | 100 (70.1-100) | 81/83 | 97.6 (91.6-99.3) |
| *Proteus vulgaris* | 5/5 | 100 (56.6-100) | | | 5/5 | 100 (56.6-100) |
| ***Salmonella* spp.** | **18/19** | **94.7 (75.4-99.1)** | **34/35** | **97.1 (85.5-99.5)** | **54/56** | **96.4 (87.9-99.0)** |
| *Salmonella* | 15/15 | 100 (79.6-100) | | | 17/17 | 100 (81.6-100) |
| *Salmonella* 4,5,12:i:- | | | 2/2 | 100 (34.2-100) | 2/2 | 100 (34.2-100) |
| *Salmonella* Heidelberg | | | 2/2 | 100 (34.2-100) | 2/2 | 100 (34.2-100) |
| *Salmonella* Infantis | | | 2/2 | 100 (34.2-100) | 2/2 | 100 (34.2-100) |
| *Salmonella* Javiana | | | 1/1 | 100 (20.7-100) | 1/1 | 100 (20.7-100) |
| *Salmonella* Montevideo | | | 7/8 | 87.5 (52.9-97.8) | 7/8 | 87.5 (52.9-97.8) |
| *Salmonella* Muenchen | | | 1/1 | 100 (20.7-100) | 1/1 | 100 (20.7-100) |
| *Salmonella* Newport | | | 6/6 | 100 (61.0-100) | 6/6 | 100 (61.0-100) |
| *Salmonella* Typhimurium | | | 7/7 | 100 (64.6-100) | 7/7 | 100 (64.6-100) |
| *Salmonella choleraesuis* subsp. *arizonae* | 0/1 | 0.0 (0.0-79.3) | | | 0/1 | 0.0 (0.0-79.3) |

| Target Species or Groups Detected by Comparator Method | PPA | | | | | |
|---|---|---|---|---|---|---|
| | Clinical Samples | | Contrived Samples | | Combined Samples | |
| | TP/TP+F N | % (95% CI) | TP/TP+F N | % (95% CI) | TP/TP+F N | % (95% CI) |
| *Salmonella enterica* subsp. *enterica* Enteritidis (Group D1) | | | 6/6 | 100 (61.0-100) | 6/6 | 100 (61.0-100) |
| *Salmonella enterica* subsp. *enterica* serovar Typhimurium | 1/1 | 100 (20.7-100) | | | 1/1 | 100 (20.7-100) |
| *Salmonella* Typhi | 2/2 | 100 (34.2-100) | | | 2/2 | 100 (34.2-100) |
| ***Serratia* spp.** | **34/34** | **100 (89.8-100)** | **36/36** | **100 (90.4-100)** | **80/80** | **100 (95.4-100)** |
| *Serratia ficaria* | | | 4/4 | 100 (51.0-100) | 4/4 | 100 (51.0-100) |
| *Serratia grimesii* | | | 3/3 | 100 (43.9-100) | 3/3 | 100 (43.9-100) |
| *Serratia liquefaciens* | | | | | 1/1 | 100 (20.7-100) |
| *Serratia marcescens* | 34/34 | 100 (89.8-100) | 19/19 | 100 (83.2-100) | 62/62 | 100 (94.2-100) |
| *Serratia plymuthica* | | | 3/3 | 100 (43.9-100) | 3/3 | 100 (43.9-100) |
| *Serratia rubidaea* | | | 7/7 | 100 (64.6-100) | 7/7 | 100 (64.6-100) |
| **Pan *Candida* spp.** | 104/110 | 94.5 (88.6-97.5) | **N/A** | **N/A** | 104/110 | 94.5 (88.6-97.5) |
| *Candida albicans* | 50/53 | 94.3 (84.6-98.1) | | | 50/53 | 94.3 (84.6-98.1) |
| *Candida glabrata* | 38/40 | 95.0 (83.5-98.6) | | | 38/40 | 95.0 (83.5-98.6) |

| Target Species or Groups Detected by Comparator Method | PPA | | | | | |
|---|---|---|---|---|---|---|
| | Clinical Samples | | Contrived Samples | | Combined Samples | |
| | TP/TP+F N | % (95% CI) | TP/TP+F N | % (95% CI) | TP/TP+F N | % (95% CI) |
| *Candida krusei* | 4/4 | 100 (51.0-100) | | | 4/4 | 100 (51.0-100) |
| *Candida parapsilosis* | 15/16 | 93.8 (71.7-98.9) | | | 15/16 | 93.8 (71.7-98.9) |
| **Pan Gram-Positive** | **628/649** | **96.8 (95.1-97.9)** | **N/A** | **N/A** | **628/649** | **96.8 (95.1-97.9)** |
| *Bacillus* (species identification not determined) | 5/8 | 62.5 (30.6-86.3) | | | 5/8 | 62.5 (30.6-86.3) |
| *Bacillus cereus* | 4/5 | 80.0 (37.6-96.4) | | | 4/5 | 80.0 (37.6-96.4) |
| *Bacillus cereus* group - not *anthracis* | 1/1 | 100 (20.7-100) | | | 1/1 | 100 (20.7-100) |
| *Enterococcus* | ½ | 50.0 (9.5-90.5) | | | 1/2 | 50.0 (9.5-90.5) |
| *Enterococcus casseliflavus* | 0/1 | 0.0 (0.0-79.3) | | | 0/1 | 0.0 (0.0-79.3) |
| *Enterococcus faecalis* | 57/61 | 93.4 (84.3-97.4) | | | 57/61 | 93.4 (84.3-97.4) |
| *Enterococcus faecium* | 23/24 | 95.8 (79.8-99.3) | | | 23/24 | 95.8 (79.8-99.3) |
| Coagulase-negative Staphylococci (CoNS) | 14/14 | 100 (78.5-100) | | | 14/14 | 100 (78.5-100) |
| *Staphylococcus* (species identification not determined) | 93/95 | 97.9 (92.6-99.4) | | | 93/95 | 97.9 (92.6-99.4) |
| *Staphylococcus aureus* | 180/182 | 98.9 (96.1-99.7) | | | 180/182 | 98.9 (96.1-99.7) |
| *Staphylococcus auricularis* | 3/3 | 100 (43.9-100) | | | 3/3 | 100 (43.9-100) |

| Target Species or Groups Detected by Comparator Method | PPA | | | | | |
|---|---|---|---|---|---|---|
| | Clinical Samples | | Contrived Samples | | Combined Samples | |
| | TP/TP+F N | % (95% CI) | TP/TP+F N | % (95% CI) | TP/TP+F N | % (95% CI) |
| *Staphylococcus capitis* | 7/7 | 100 (64.6-100) | | | 7/7 | 100 (64.6-100) |
| *Staphylococcus cohnii* | 2/2 | 100 (34.2-100) | | | 2/2 | 100 (34.2-100) |
| *Staphylococcus epidermidis* | 89/91 | 97.8 (92.3-99.4) | | | 89/91 | 97.8 (92.3-99.4) |
| *Staphylococcus haemolyticus* | 8/8 | 100 (67.6-100) | | | 8/8 | 100 (67.6-100) |
| *Staphylococcus hominis* | 23/23 | 100 (85.7-100) | | | 23/23 | 100 (85.7-100) |
| *Staphylococcus hominis subsp. hominis* | 21/21 | 100 (84.5-100) | | | 21/21 | 100 (84.5-100) |
| *Staphylococcus intermedius* | 1/1 | 100 (20.7-100) | | | 1/1 | 100 (20.7-100) |
| *Staphylococcus lugdunensis* | 1/1 | 100 (20.7-100) | | | 1/1 | 100 (20.7-100) |
| *Staphylococcus saccharolyticus* | 1/1 | 100 (20.7-100) | | | 1/1 | 100 (20.7-100) |
| *Staphylococcus saprophyticus* | 1/1 | 100 (20.7-100) | | | 1/1 | 100 (20.7-100) |
| *Staphylococcus sciuri* | 1/1 | 100 (20.7-100) | | | 1/1 | 100 (20.7-100) |
| *Staphylococcus simulans* | 2/2 | 100 (34.2-100) | | | 2/2 | 100 (34.2-100) |
| *Staphylococcus warneri* | 4/4 | 100 (51.0-100) | | | 4/4 | 100 (51.0-100) |
| Alpha-hemolytic *Streptococcus* spp. | 1/1 | 100 (20.7-100) | | | 1/1 | 100 (20.7-100) |

| Target Species or Groups Detected by Comparator Method | PPA | | | | | |
|---|---|---|---|---|---|---|
| | Clinical Samples | | Contrived Samples | | Combined Samples | |
| | TP/TP+F N | % (95% CI) | TP/TP+F N | % (95% CI) | TP/TP+F N | % (95% CI) |
| Beta-hemolytic streptococci, Group G | 1/1 | 100 (20.7-100) | | | 1/1 | 100 (20.7-100) |
| Gamma-hemolytic *Streptococcus* spp. | 1/1 | 100 (20.7-100) | | | 1/1 | 100 (20.7-100) |
| *Streptococcus* (species identification not determined) | 9/10 | 90.0 (59.6-98.2) | | | 9/10 | 90.0 (59.6-98.2) |
| *Streptococcus* - viridans group | 18/19 | 94.7 (75.4-99.1) | | | 18/19 | 94.7 (75.4-99.1) |
| *Streptococcus agalactiae* | 21/21 | 100 (84.5-100) | | | 21/21 | 100 (84.5-100) |
| *Streptococcus anginosus* | 2/2 | 100 (34.2-100) | | | 2/2 | 100 (34.2-100) |
| *Streptococcus anginosus group* | 5/6 | 83.3 (43.6-97.0) | | | 5/6 | 83.3 (43.6-97.0) |
| *Streptococcus bovis* | 2/2 | 100 (34.2-100) | | | 2/2 | 100 (34.2-100) |
| *Streptococcus constellatus* subsp. *constellatus* | 1/1 | 100 (20.7-100) | | | 1/1 | 100 (20.7-100) |
| *Streptococcus dysgalactiae* (Group G) | 4/4 | 100 (51.0-100) | | | 4/4 | 100 (51.0-100) |
| *Streptococcus gordonii* | 1/1 | 100 (20.7-100) | | | 1/1 | 100 (20.7-100) |
| *Streptococcus infantarius* | 1/1 | 100 (20.7-100) | | | 1/1 | 100 (20.7-100) |
| *Streptococcus intermedius* | 1/1 | 100 (20.7-100) | | | 1/1 | 100 (20.7-100) |
| *Streptococcus mitis* | 11/12 | 91.7 (64.6-98.5) | | | 11/12 | 91.7 (64.6-98.5) |

| Target Species or Groups Detected by Comparator Method | PPA | | | | | |
|---|---|---|---|---|---|---|
| | Clinical Samples | | Contrived Samples | | Combined Samples | |
| | TP/TP+F N | % (95% CI) | TP/TP+F N | % (95% CI) | TP/TP+F N | % (95% CI) |
| *Streptococcus mitis group* | 3/3 | 100 (43.9-100) | | | 3/3 | 100 (43.9-100) |
| *Streptococcus oralis* | 1/1 | 100 (20.7-100) | | | 1/1 | 100 (20.7-100) |
| *Streptococcus pneumoniae* | 23/23 | 100 (85.7-100) | | | 23/23 | 100 (85.7-100) |
| *Streptococcus pyogenes* | 9/9 | 100 (70.1-100) | | | 9/9 | 100 (70.1-100) |
| *Streptococcus salivarius* | 3/3 | 100 (43.9-100) | | | 3/3 | 100 (43.9-100) |

**Supplemental Table 4: GN Clinical Study: Organisms Identified by Standard-of-Care Methods that are not Targeted by the BCID-GN (Prospective/Retrospective Samples)**

| **Organism Type** | **Off-Panel Organism Identified by SOC** | **Count** |
|---|---|---|
| Gram positive | Aerobic Gram-Positive Bacilli/No further identification | 1 |
| Gram positive | *Aerococcus viridans* | 1 |
| Gram positive | *Clostridium perfringens* | 1 |
| Gram positive | *Clostridium* species | 1 |
| Gram positive | *Cutibacterium acnes (P. acnes)* | 1 |
| Gram positive | *Lactococcus lactis* | 1 |
| Gram positive | *Micrococcus luteus* | 2 |
| Gram positive | *Peptostreptococcus anaerobius* | 1 |
| Gram positive | *Propionibacterium* | 1 |
| Gram negative | *Achromobacter* species | 1 |
| Gram negative | *Achromobacter xylosoxidans* | 1 |
| Gram negative | *Achromobacter xylosoxidans* subsp. *xylosoxidans* | 1 |

| | | |
|---|---|---|
| Gram negative | *Acinetobacter* | 1 |
| Gram negative | *Acinetobacter baumannii complex (baum-calcoac-13TU)* | 1 |
| Gram negative | *Acinetobacter lwoffii* | 1 |
| Gram negative | *Acinetobacter radioresistens* | 3 |
| Gram negative | *Aeromonas caviae* | 1 |
| Gram negative | *Aeromonas veronii* | 1 |
| Gram negative | *Aggregatibacter actinomycetemcomitans* | 1 |
| Gram negative | *Bacteroides ovatus* | 1 |
| Gram-negative | *Bacteroides thetaiotaomicron* | 1 |
| Gram negative | *Burkholderia cepacia* complex | 1 |
| Gram negative | *Campylobacter jejuni* | 1 |
| Gram negative | *Citrobacter amalonaticus* | 1 |
| Gram negative | *Enterobacteriaceae,* unable to further speciate | 1 |
| Gram negative | *Kluyvera* species | 1 |
| Gram negative | *Leclercia adecarboxylata* | 2 |
| Gram negative | *Moraxella osloensis* | 1 |
| Gram negative | *Moraxella* species | 1 |

| | | |
|---|---|---|
| Gram negative | *Pantoea* species | 1 |
| Gram negative | *Prevotella* species | 1 |
| Gram negative | *Providencia stuartii* | 6 |
| Gram negative | *Pseudomonas putida* | 1 |
| Gram negative | *Roseomonas* species | 1 |
| Gram negative | *Sphingomonas (Pseudomonas) paucimobilis* | 2 |
| Gram negative | *Wolinella species* | 1 |
| Gram negative | Non-fermenting Gram-negative bacilli | 1 |
| *Candida* spp. | *Candida lusitaniae* | 1 |

44 of 926 Samples Tested

Total Gram-positive missed = 9

Total Gram-negative missed = 33
